# A Novel Method for Estimating Chlorophyll and Carotenoid Concentrations in Leaves: A Two Hyperspectral Sensor Approach

**DOI:** 10.3390/s23083843

**Published:** 2023-04-09

**Authors:** Renan Falcioni, Werner Camargos Antunes, José Alexandre Melo Demattê, Marcos Rafael Nanni

**Affiliations:** 1 Department of Agronomy, State University of Maringa, Av. Colombo, 5790, Maringa 87020-900, Parana, Brazil; wcantunes@uem.br (W.C.A.); mrnanni@uem.br (M.R.N.); 2 Department of Soil Science, Luiz de Queiroz College of Agriculture, University of Sao Paulo, Av. Padua Dias, 11, Piracicaba 13418-260, Sao Paulo, Brazil; jamdemat@usp.br

**Keywords:** cellular structures, chlorophyll and carotenoids, leaf optical properties, leaf thickness, partial least squares regression, transmission electron microscopy

## Abstract

Leaf optical properties can be used to identify environmental conditions, the effect of light intensities, plant hormone levels, pigment concentrations, and cellular structures. However, the reflectance factors can affect the accuracy of predictions for chlorophyll and carotenoid concentrations. In this study, we tested the hypothesis that technology using two hyperspectral sensors for both reflectance and absorbance data would result in more accurate predictions of absorbance spectra. Our findings indicated that the green/yellow regions (500–600 nm) had a greater impact on photosynthetic pigment predictions, while the blue (440–485 nm) and red (626–700 nm) regions had a minor impact. Strong correlations were found between absorbance (R^2^ = 0.87 and 0.91) and reflectance (R^2^ = 0.80 and 0.78) for chlorophyll and carotenoids, respectively. Carotenoids showed particularly high and significant correlation coefficients using the partial least squares regression (PLSR) method (R^2^_C_ = 0.91, R^2^cv = 0.85, and R^2^_P_ = 0.90) when associated with hyperspectral absorbance data. Our hypothesis was supported, and these results demonstrate the effectiveness of using two hyperspectral sensors for optical leaf profile analysis and predicting the concentration of photosynthetic pigments using multivariate statistical methods. This method for two sensors is more efficient and shows better results compared to traditional single sensor techniques for measuring chloroplast changes and pigment phenotyping in plants.

## 1. Introduction

Leaf optical properties, such as reflectance, absorbance, and transmittance, are influenced by a variety of factors, including the type and amount of pigments present in the leaf, as well as their distribution within cellular structures such vacuoles and chloroplasts [1,2,3,4,5,6]. Higher concentrations of photosynthetic pigments result in increased light absorption and decreased reflectance in the visible spectrum [7,8]. Environmental factors, such as light intensity and plant hormones, can also impact leaf optical properties and lead to changes in parameters such as leaf thickness, specific leaf area, leaf area index, cell architecture, and biochemical composition [6,7,9,10]. For example, supplementing with gibberellin (GA_3_) leads to thinner leaves, while inhibiting gibberellin biosynthesis with paclobutrazol (PAC) results in smaller, darker green plants with thicker leaves, leading to changes in the optical profile and spectral leaf signatures [8].

Efforts have been made to predict the presence of leaf pigments using sensors by remote sensing techniques [6,11]. Chlorophylls significantly impact leaf spectral patterns, as they have two major peak absorptions, with blue peaks at 430 and 453 nm for chlorophyll *a* and *b*, respectively, and red peaks at 642 and 662 nm for chlorophyll *b* and *a*, respectively [12]. Carotenoids have a broad absorption range in blue (400–500 nm), which overlaps with the chlorophyll spectrum. In this way, nondestructive analysis models have been developed to estimate these pigments [13,14,15]. However, some researchers overlook the impact of changes in the leaf profile and opt for selecting specific bands in the infrared region instead of contiguous hyperspectral bands in the visible spectrum, which can produce robust models but lack information about in vivo systems, such as biochemical structures and leaf spectral signatures [8]. Accordingly, Yacobi (2012) estimated chlorophyll *a* using the inverse of the complete reflectance spectrum, but not chlorophyll *b*, as blue bands (400–470 nm) were not considered [16]. However, Jin et al. (2022) [17] suggested that specific mid-infrared bands could estimate the total chlorophyll concentration in tobacco plants, but this approach neglects the visible region and results in a limited understanding of pigment prediction and photosynthetic activity [16,17,18].

Multivariate statistical methods applied to hyperspectral data analysis provide a valuable and precise understanding of various plant factors, such as productivity, nutrient imbalances, temperature stress, changes in the xanthophyll cycle, and mesophyll cell structure [10,19,20]. In this sense, new methods are being developed to monitor leaf pigment accumulation during plant growth and development, and spectral reflectance is one of the most widely used techniques, although it may not always be reliable due to the overlapping spectra of different pigments and intrinsic variations in leaf structure [21,22,23]. To overcome these limitations, the use of spectral absorption has shown promising results in the remote sensing of leaf pigments [21,22,24]. In a study by Gitelson and Solovchenko (2018) [25], the authors found excellent results by analyzing the transmittance data collected from a Virginia creeper and transforming it into absorbance using the equation A = −ln(T). This approach, which combines leaf absorption profile data from hyperspectral sensors with other leaf characteristic data, offers a more comprehensive understanding of the in vivo optical system, thereby enhancing the analysis of leaf pigments compared to traditional destructive analyzes involving spectrophotometry, chromatography, or spectroscopy methods [6,11].

As reported by [24,25,26], PLSR has become a popular multivariate statistical technique for improving predictions based on leaf optical properties [27]. PLSR has been shown to have a strong relationship with the prediction of photosynthetic pigment concentration, especially when combined with multispectral and hyperspectral data [5,28]. PLSR, when combined with remote sensing techniques that collect high-resolution data across various wavelengths [28,29], offers a powerful tool to understand the intricate relationships between plant physiology, environmental conditions, and the prediction of various biochemical and biophysical process parameters [5,23,30]. These combined technologies can improve predictions of plant growth, stress response, and pigment phenotypic variation in agronomically and ecologically significant plant species [14,30,31,32,33]. Furthermore, this approach can reveal insights into the organization of mesophyll and the ultrastructure of chloroplasts and thylakoid membranes, enhancing our understanding of plant physiology.

**Figure 1:**
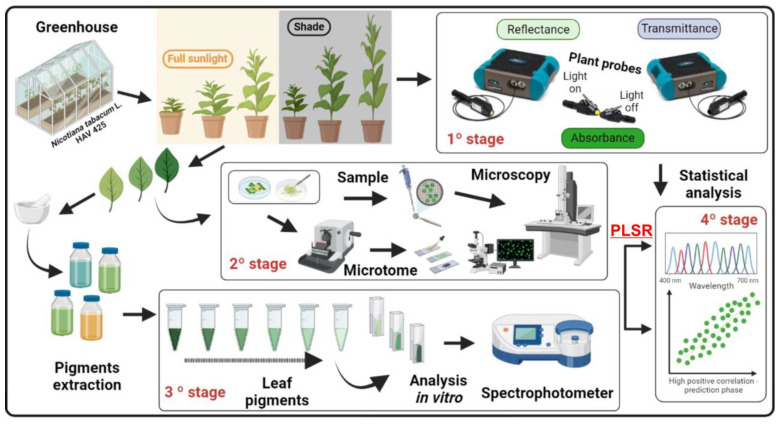
Flowchart describing the prediction of chlorophylls and carotenoids in *Nicotiana tabacum* L., using one or two hyperspectral sensors. Plants are grown in a greenhouse under full sunlight or shading (**1° stage**), and their leaves are measured using single or two hyperspectral sensors. (**2° stage**), the leaves are analyzed using light and transmission electron microscopy. (**3° stage**), the pigments are quantified using classical destructive analysis with a spectrophotometer. (**4° stage**), the data are analyzed using PLS statistics.

The objective of this study was to determine the total concentration of chlorophylls *a*, chlorophyll *b*, chlorophyll total (*a*+*b*), and carotenoids in tobacco plants grown under full sunlight and shade conditions using a new and improved method of analysis. Our hypothesis is that this can be achieved through the analysis of both reflectance (R) and transmittance (T) spectral curves in the visible region (400–700 nm) obtained from two hyperspectral sensors and the subsequent conversion of the data to absorbance (A) using the equation A = 1 − (R + T). This approach integrates the effects of the light environment and pigments to enhance our understanding of leaf pigment concentrations through the use of transmission electron microscopy (TEM) and evaluate the distribution and organization of pigments inside the chloroplast. Additionally, this method is noninvasive and provides a more accurate representation of the in vivo plant system than traditional destructive methods based on PLSR statistics following the flowchart proposed in Figure 1.

## 2. Materials and Methods

### 2.1. Plant Material and Experimental Design

**Figure 2:**
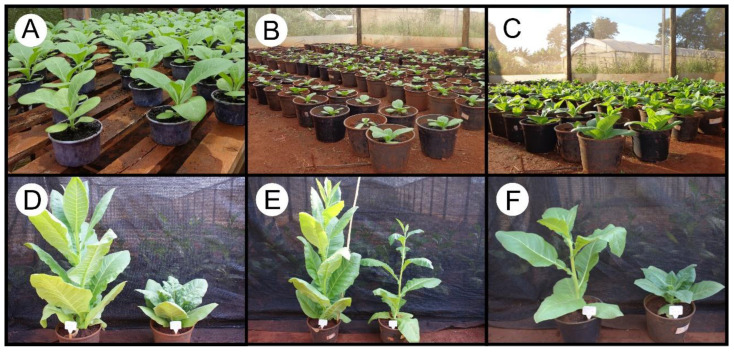
Representative images of tobacco plants with variations in phenotype. (**A**–**C**) Differentiated growth stages of tobacco plants in the greenhouse. (**D**–**F**) Effects of gibberellic acid (GA_3_; elongated plants) or paclobutrazol (PAC, shorter plants) on the growth plants under different light conditions (high and low light, normal and etiolated phenotypes).

Spectroradiometric monitoring was conducted on *Nicotiana tabacum* L. cv. Havana 425 plants grown in a greenhouse at the Technological Center for Irrigation of the State University of Maringá (CTI-UEM). Seedlings with 5–6 expanded leaves, measuring over 5 cm in length, were selected for their uniformity, health, and vigor. These seedlings were then transplanted into 5 L plastic pots filled with medium-textured soil and fertilized with N, P, and K (10-10-10). The leaves of the plants, which had varying ages and levels of gibberellins (GA_3_) and paclobutrazol (PAC), were evaluated over a 20-day period (Figure 1 and Figure 2). In addition, two different light intensities were used, i.e., full (100%) sunlight irradiation and low light (8.5% of sunlight), to investigate their chlorophylls and carotenoids pigment concentration parameters, which were evaluable by single and two sensors by spectroradiometers.

### 2.2. Extraction of Leaf Pigments

The method for extracting leaf pigments involved using 2 cm^2^ aluminum standard molds to obtain samples from young, healthy, and expanded leaves. The samples were crushed and then immersed in 10 mL of an 80% acetone-water solution for 18–20 h in the dark. The extracted samples were then subjected to absorption spectra measurement using a Lambda 1050 UV/VIS/NIR spectrophotometer (PerkinElmer, Inc.,Hopkinton, MA, USA). The concentration of chlorophylls (*a*, *b*, and total (*a*+*b*)) and carotenoids (carotenes and xanthophylls) were determined using the equations reported by Lichtenthaler [34] (mg L^−1^) and expressed per unit area of leaf:

Chlorophyll *a* = Chl *a* = 12.25* × λ663 nm − 2.79* × λ646 nm (1)

Chlorophyll *b* = Chl *b* = 21.50* × λ646 nm − 5.10* × λ663 nm (2)

Chlorophyll total = Chl (*a*+*b*) = 7.15* × λ663 nm + 18.71* × λ646 nm (3)

Carotenoids = (1000* × λ470 nm − 1.82* × Chl *a* − 85.02* × Chl *b*)/198 (4)

λ: wavelength selected in a spectrophotometer;

*: coefficient of equations;

Pigment concentrations expressed as per unit area of leaf can be calculated using the following general formula, which takes into account the pigment concentration in the extraction solution, the volume of the extraction solution, and the leaf area:

(5) $$Pigment\;\left({mg\;m}^{-2}\right)=\frac{\left(\frac{Equation\;of\;pigment({mg\;L}^{-1})\;\times \;Volume\;extraction\;\left(mL\right)\;\times \;Fd}{1000}\right)}{Leaf\;area\;(m^{2})}$$

where:

Equations for the determinations of pigments in acetone (80%, *v*/*v*) (Equations (1)–(4)): Chlorophyll *a*, Chlorophyll *b*, Chlorophyll total, Carotenoids;

Volume extraction (mL): 10 mL or volume immersed segment of leaves for extraction;

Fd: Factor of dilution if necessary; considering the 0 to 1 range for absorbance of solution;

Leaf area (m^2^) = segment of area extraction; conversion cm^2^ to m^2^; factor of 10,000

1000 = Factor of conversion of the equation;

Thus, pigments can be expressed in mg m^−2^ or g m^−2^.

### 2.3. Optical Microscopy Analysis

To analyze the leaf samples, 2 cm^3^ of the medial region was fixed with Karnovsky’s solution and stored at 4 °C. The samples were then dehydrated with an ethanol series (50%, 70%, 80%, 90%, and 100% (repeated three times)) and infiltrated with methyl methacrylate (Leica Historesin^®^). Sectioning was conducted using a rotation microtome (Eikonal, São Paulo, SP, BRA), and the 8 μm sections obtained were stained with toluidine blue in acetate buffer at pH 4.7. The images were captured using a Leica ICC50 light microscope (Leica Company, Wetzlar, DEU). The qualitative analysis of the microscopic images was used to investigate the structural organization, cellular arrangement, and overall organization of the leaf mesophyll.

### 2.4. Transmission Electron Microscopy

The transverse sections of the leaf samples were analyzed using transmission electron microscopy (TEM). After fixing the samples in a modified Karnovsky solution in a 0.05 M cacodylate buffer (pH 7.2) and postfixed with 1% osmium tetroxide for 1 h, they were contrasted in a 0.5% uranyl acetate solution overnight. The samples were then dehydrated in an acetone series (30%, 50%, 70%, 80%, 90%, and 100% repeated three times), infiltrated with Spurr low viscosity epoxy resin, and sliced into 70 nm sections. The sections were contrasted with 3% uranyl acetate and lead citrate solutions and analyzed using a JEOL JEM 1400 TEM (Leica Microsystems Inc., Peabody, IL, USA) at 80 kV. The organization of chloroplasts and electron density of thylakoid membranes were analyzed using ultrastructural images.

### 2.5. Hyperspectral Optical Leaf Properties

**Figure 3:**
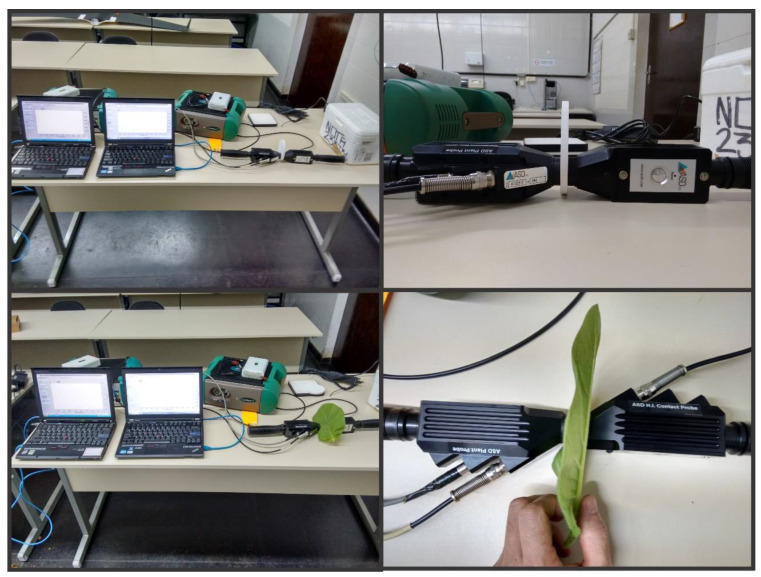
A representative image displays two spectroradiometers for the simultaneous collection of reflectance and transmittance data. (**Top**) the spectroradiometers were calibrated with a Spectralon^®^. (**Bottom**) data were collected from *Nicotiana tabacum* leaves using Plant Probes.

The reflectance (R) and transmittance (T) of leaves were collected using a FieldSpec^®^ spectroradiometer 3 (Analytical Spectral Devices ASD Inc., Longmont, CO, USA) coupled to an ASD contact PlantProbe^®^ probe with a 10 mm diameter. The spectroradiometer had used detection sensors: 512 Si photodiodes capturing wavelengths from 350–1000 nm. To ensure the data were free of atmospheric effects, a PlantProbe^®^ leaf clip (Analytical Spectral Devices ASD Inc; USA) was used. Standard white reference plates (Spectralon^®^, Labsphere Inc., Longmont, CO, USA) were used for equipment calibration and optimization. A high-intensity light beam (over 2000 µmol m^−2^ s^−1^) from a plant probe of the spectroradiometer was directed onto the adaxial surface of the leaves, while a second plant probe with the light beam off was positioned to measure the abaxial surface of the leaves. Both the reflectance and transmittance were measured simultaneously for each wavelength. The equipment was set to average 50 readings per sample to produce a spectral curve. Absorbance (A) was calculated as [A = 1 − (R + T)] [6,8]. Data from the pigment concentration for the photosynthetically active region (400–700 nm) of the leaves were used in the study. Additional information can be found in Figure 1 and Figure 3.

### 2.6. Data Processing

The statistical model was developed using 150 samples, which were randomly divided into two groups: the first set comprised 100 samples used for calibration and cross-validation, and the second group consisted of 50 independent samples used for external prediction of the PLSR model. Chlorophyll *a*, chlorophyll *b*, chlorophyll *a*+*b*, and carotenoids were compared to the spectral curves, with each variable being treated as an independent variable. The multivariate calibration models were developed using PLSR with the Nonlinear Iterative Partial Least Squares (NIPALS) algorithm, and output outlier limits were defined by Leverage’s type and analyzed by Leverage and Hotelling’s T^2^ (limit at 5%). The predictive ability of the calibration models was evaluated by calculating the coefficients of determination (R^2^) and the root mean square error (RMSE_C_ for calibration, RMSE_CV_ for cross-validation, and RMSE_P_ for prediction phases). The leave-one-out cross-validation method was used as a preliminary form of attribute prediction, with an independent predictor based on an unknown data set also used in parallel. According to Minasny and McBratney (2013) [35], R^2^ > 0.75 values are considered to display excellent prediction capacity, R^2^ values between 0.75 and 0.5 are considered good, and R^2^ < 0.5 is considered low. Additionally, the ratio of performance to deviation (RPD) was calculated using the equation $RPD=\frac{1}{\sqrt{{1-R}^{2}}}$ with R^2^ calculation (R^2^_C_), cross-validation (R^2^_CV_), and predicted (R^2^_P_) for the calculation and applied as a useful indicator of the expected accuracy of PLS predictions. For a quality analytical performance, RPD must be at least 3 for agricultural applications, while RPDs between 2 and 3 are considered good, 1.5–2 as medium, and lower than 1.5 as poor [36]. The β-coefficients, Ŷ = +β0 + β1λ1 + … + βnλn + ε, for pigment parameters obtained with hyperspectral data for reflectance and absorbance curves (400 at 700 nm) are displayed. All statistical analyzes were performed using the following software packages: The Unscramber x10.4^®^ (Camo Software, Oslo, Norway), Statistica 12.0^®^ (Statsoft Inc., Uppsala, Sweden), SigmaPlot 12.0^®^ (Systat Inc., San Jose, CA, USA), and CorelDraw 2020^®^ (Corel Corp., Ottawa, ON, Canada).

## 3. Results and Discussion

### 3.1. Structure, Ultrastructure, and Photosynthetic Pigments

**Figure 4:**
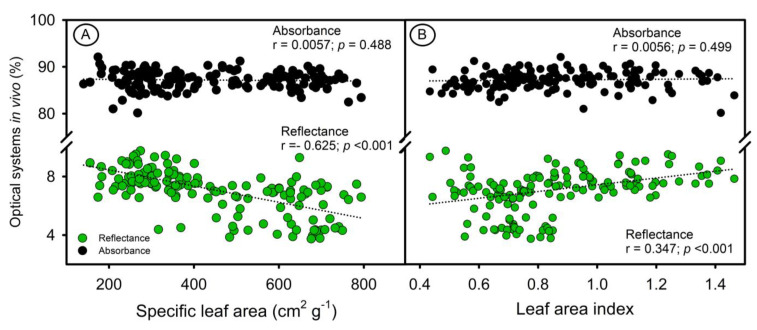
Correlation between reflectance and absorbance in optical systems in vivo. (**A**) Specific leaf area. (**B**) Leaf area index.

The morpho-anatomical characteristics of leaves, influenced by GA_3_, PAC, or light, impact the total leaf area, specific leaf area (SLA), and leaf area index (LAI), which in turn affect the absorbance spectra [2,6,37]. However, there were only slight variations in the reflectance spectra in bands where chlorophylls had strong absorption [2,8,38]. Accordingly, Hogewoning et al. (2012) [38] and Falcioni et al. (2017) [8] showed that leaf appearance (reflectance) and the contribution of absorbance (light leaves with low absorbance vs. dark leaves with high absorbance) in the leaf optical system (Figure 4) were related to the concentration of pigments in *N. tabacum* plants.

**Figure 5:**
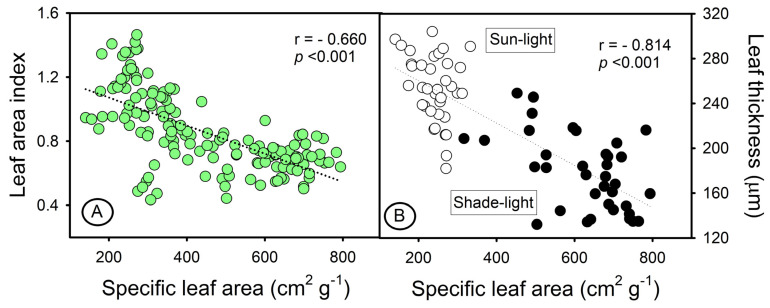
Correlations between intrinsic parameters and the influence of leaf optical properties. (**A**) Specific leaf area (SLA) and leaf area index (LAI). (**B**) Specific leaf area and leaf thickness in sunlight and shade light. Green dots show total plants; white sunlight plants; black shade-light plants.

Variations in structural components such as leaf thickness, changes in SLA and LAI, and ultrastructural changes (larger and denser chloroplasts and thylakoids) led to increased light absorption and changed the optical properties of the leaf. Reflectance spectroscopy showed significant correlations with SLA and LAI (*p* < 0.001), but no correlations were found between absorbance and these parameters (*p* = 0.488 and 0.499, respectively) (Figure 4). On the other hand, the intrinsic properties of the structural factor associated with the interaction of light with cell walls, leaf thickness, and parenchyma layers were highlighted, but not necessarily with the concentration of leaf pigments. Furthermore, reflectance hyperspectral data can provide parameter estimates and show significant correlations, but do not fully reveal the interaction of pigments in the electromagnetic spectrum when the absorbance in two sensors was measured in the in vivo leaf system [10] (Figure 4 and Figure 5).

**Figure 6:**
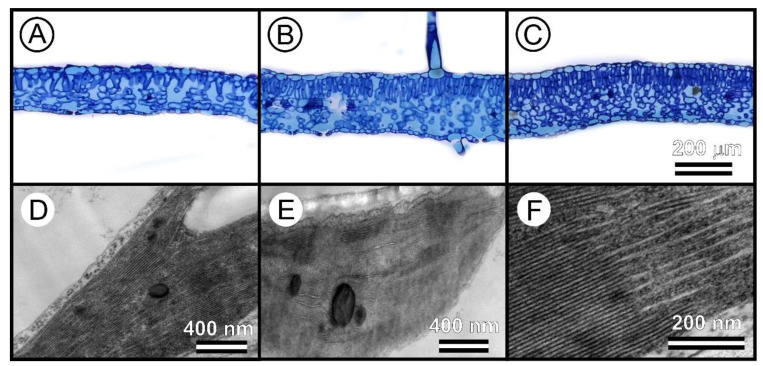
Microscopy of leaves. (**A**–**C**) Light microscopy. (**D**–**F**) Electron transmission microscopy showed the characteristics of the chloroplasts and thylakoids in tobacco leaves. Note the leaf thickness and electron density of thylakoid membrane changes. Scale bars = 200 µm, 400 nm, and 200 nm.

Tobacco leaves have a mesophyll that is composed of both palisade and spongy parenchyma cells [8]. In this way, light and GAs levels impacted the thickness of the leaves, altering SLA and LAI (Figure 4 and Figure 5). Furthermore, the interaction of light with the pigments in the mesophyll tissue, cell wall, and chloroplasts was also related by [6,8,10]. Accordingly, Falcioni et al. (2017) [8] palisade parenchyma cells, which are 1 to 3 layers thick, can become more overlapped, increasing SLA (r = −0.66) and reducing leaf thickness (r = −0.84), which can affect the estimation of photosynthetic pigments based on absorbance. Therefore, integrating leaf optical properties with reflectance data can provide a more accurate representation of the in vivo system (Figure 6), as reported in [6,8,24].

The structural components and optical properties of tobacco leaves can be influenced by environmental factors and plant hormones, including but not limited to light intensity, growth inhibitors, GA_3_, cytokinins, and ascorbic acid. These factors can result in changes in leaf thickness (Figure 6A–C), the number of parenchyma layers, and chloroplast ultrastructure [8,10]. The arrangement and amount of chloroplasts and thylakoids have a significant impact on the green/yellow region (500–600 nm) of the leaf’s optical properties [10,39]. However, the blue (440–485 nm) and red (626–700 nm) regions of the spectrum are less impacted and not statistically significant (*p* > 0.05) [8,10].

These findings, confirmed by transmission microscopy (Figure 6D–F), emphasize the importance of considering the role of sensors in measuring the optical properties of leaves to accurately represent leaf behavior in vivo.

**Figure 7:**
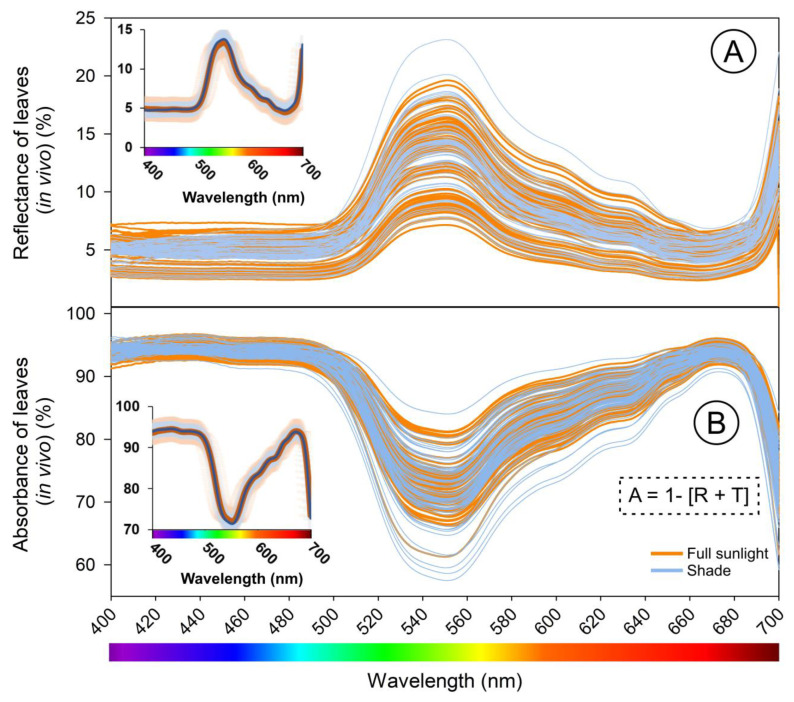
Spectral leaf reflectance and absorbance (in vivo) curve from 400 to 700 nm in *Nicotiana tabacum* leaves of plants grown in a greenhouse with different growth stages and contents of gibberellin acid (GA_3_) or with paclobutrazol (PAC) and full sunlight and shade. (**A**) The reflectance curves were obtained with one of the spectroradiometers. (**B**) The absorbance curve was obtained by equation [A = 1 − (R + T)] (more details in Figure 1 and Figure 2). Insets on the left show the mean ± SE. Orange lines tobacco grown full sunlight and blue lines shade. (*n* = 150).

The reflectance index of hyperspectral curves has been found to be negatively correlated with chlorophyll concentration, leaf thickness, and elongated parenchymatic cells [8,37]. For example, higher levels of PAC can result in a higher number of parenchymal cells and an accumulation of chlorophylls, leading to darker leaves with lower reflectance values but higher absorbance. On the other hand, leaves grown in shaded environments have a lighter color, more chlorophyll by mass, higher reflectance, and lower absorbance (Figure 7). In this sense, leaves grown under high light conditions show higher levels of absorbance than those grown under low light conditions [8,40,41,42]. Therefore, changes in leaf structure and ultrastructure can affect leaf absorption spectra and have a stronger correlation with leaf optical properties and the estimation of photosynthetic pigments compared to the reflective characteristics of the epidermis (Figure 7) or following reported estimates obtained from reflectance data [18,43]. Additionally [8,41], reported that the peaks and valleys at 435 nm, 550 nm, and 674 nm can be influenced by pigment levels, such as chlorophylls, carotenoids, and hormone content that regulates phenotypic plasticity in response to growth and environmental conditions [8,41].

**Figure 8:**
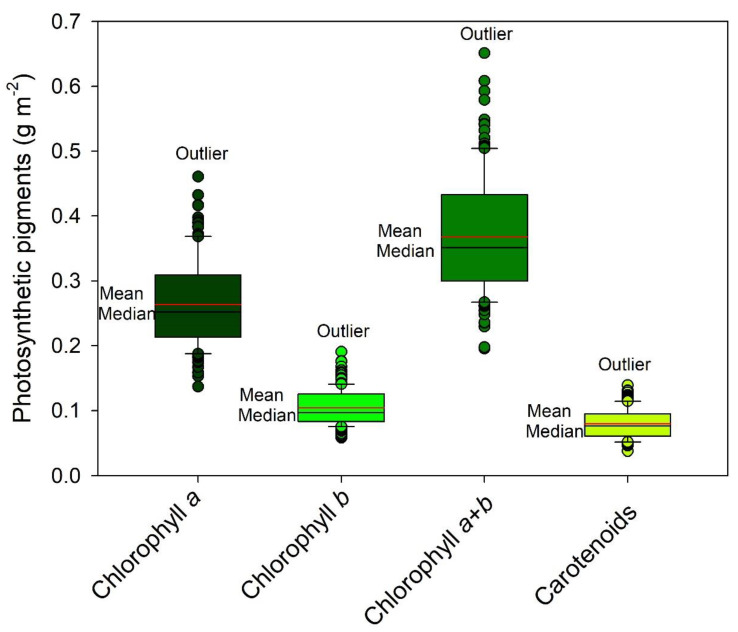
Box plot of descriptive statistics of photosynthetic pigments (chlorophylls *a*, *b*, total (*a*+*b*) and carotenoids) quantified from the leaves of *Nicotiana tabacum* in which the reflectance (R), transmittance (T), and absorbance (A) spectra were measured. (*n* = 150).

The confinement of pigments in tobacco leaves remains uncertain when measured by a single sensor [10]. The thylakoid membranes (Figure 6D–F) were found to mainly impact the green spectral band (525–580 nm) but not necessarily the blue and red bands [10]. For example, variations in pigments (Figure 8) were observed, but not in leaf thickness (Figure 7 and Figure 8). To overcome this limitation, using two sensors to measure the optical properties of leaves in crop plants [44], quantify pigment concentration [30], or monitor the growth and development of pigments in leaves may be a more effective alternative to traditional methods of analysis (Figure 1 and Figure 3).

### 3.2. Reflectance and Absorbance Model for Photosynthetic Pigment Prediction

#### 3.2.1. Calibration Models

**Figure 9:**
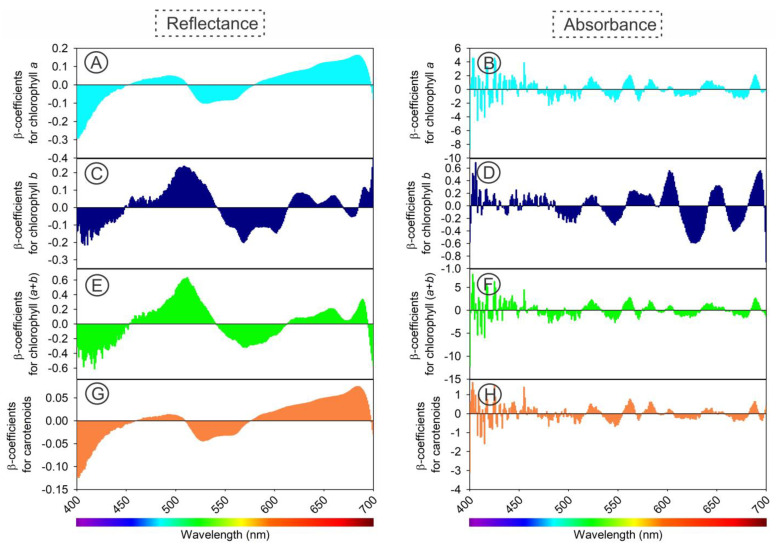
Regression coefficients of the PLSR model for (**A**,**B**) chlorophyll *a* (Chl *a*), (**C**,**D**) chlorophyll *b* (Chl *b*), (**E**,**F**) chlorophyll *a*+*b* (Chl *a*+*b*), and (**G**,**H**) carotenoids (Car). Reflectance (**A**,**C**,**E**,**G**) and absorbance (**B**,**D**,**F**,**H**) data. (*n* = 150).

**Figure 10:**
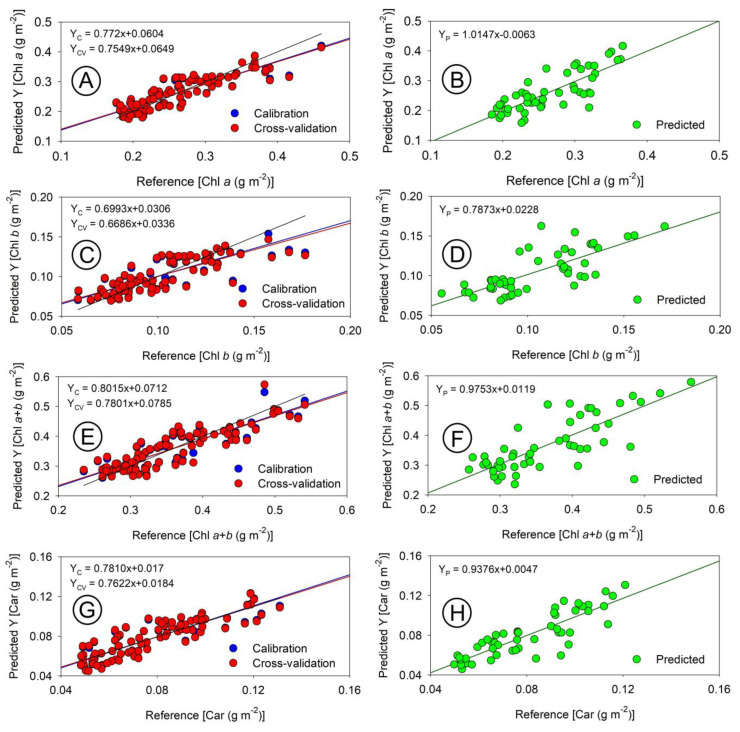
Scatterplot of models based on reflectance curves (400–700 nm) obtained with the spectroradiometer. (**A**,**B**) chlorophyll *a* (Chl *a*). (**C**,**D**) chlorophyll *b* (Chl *b*). (**E**,**F**) chlorophyll total (Chl *a*+*b*). (**G**,**H**) carotenoids (Car). Linear equation estimates were reported for calibration (Y_C_; blue dots), cross-validation (Y_CV_; red dots), and predicted (Y_P_; green dots).

**Figure 11:**
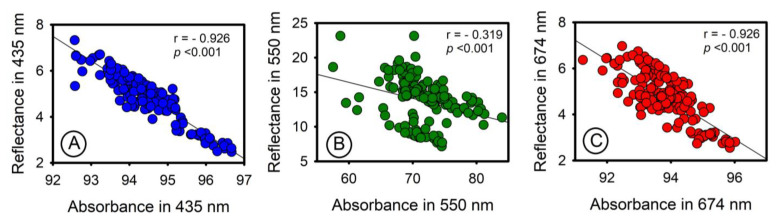
Pearson’s correlation between absorbance and reflectance specific points of the blue, green, and red bands selected in leaves. (**A**) Wavelength at 435 nm. (**B**) Wavelength at 550 nm. (**C**) Wavelength at 674 nm.

**Figure 12:**
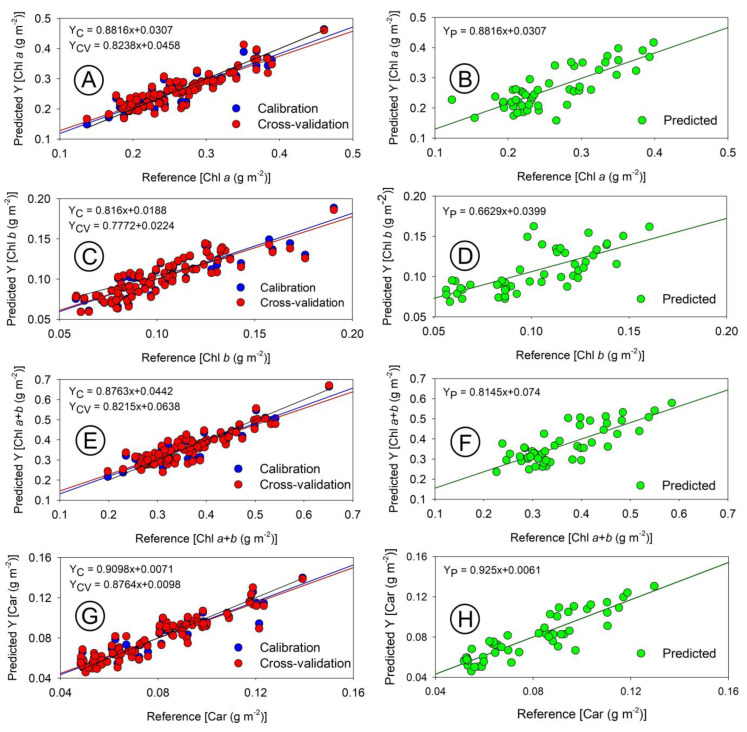
Scatterplot of models based on absorbance curves (400–700 nm) obtained with the spectroradiometer. (**A**,**B**) chlorophyll *a* (Chl *a*). (**C**,**D**) chlorophyll *b* (Chl *b*). (**E**,**F**) chlorophyll total (Chl *a*+*b*). (**G**,**H**) carotenoids (Car). Linear equation estimates were reported for calibration (Y_C_; blue dots), cross-validation (Y_CV_; red dots), and predicted (Y_P_; green dots).

Utilizing hyperspectral sensors, we applied a comparative method to estimate the concentration of photosynthetic pigments (Figure 9, Figure 10, Figure 11 and Figure 12). Our calibration models, built using PLSR for chloroplast pigments (chlorophyll *a*, chlorophyll *b*, total chlorophyll (*a*+*b*), and carotenoids), showed better results when using absorbance data compared to reflectance data (Table 1 and Figure 9, Figure 10, Figure 11 and Figure 12). The statistical parameters used in the evaluation revealed that despite significant variation in plant growth due to anatomical and ultrastructural changes, we were able to obtain analytical data that closely matched the results obtained through spectrophotometric pigment concentration (Figure 8). For example, refs. [6] and [17,45] both reflectance data (Figure 10A–H) and absorbance data (Figure 12A–H), as well as the predictive results (Figure 9, Figure 10 and Figure 12), demonstrated the accuracy of the multivariate calibration approach.

The highest coefficients of determination were obtained for carotenoids (Car) and the sum of chlorophyll *a* and *b* (Chl *a*+*b*) for both reflectance and absorbance data (Figure 9A–F). For example, [46] reported the lowest values were obtained for estimating chlorophyll *b* (Chl *b*), but all values were still significantly higher (*p* < 0.05). The results showed that absorbance data estimated chloroplast pigments, particularly carotenoids, more accurately than reflectance data (Figure 10C,D). In this way, improved accuracy could be due to the more efficient collection of absorbance data using two hyperspectral sensors. The study also found that reflectance data, although similar to the results reported by Jin and Wang (2019) [47] with R^2^ ≥ 0.77, showed even greater values (R^2^ ≥ 0.88) with the proposed new method. Previous research by Gitelson and Solovchenko (2018) [25] on *P. quinque-folia* demonstrated high values (R^2^ ≥ 0.92) for chlorophylls when reflectance data collected in an integrating sphere were transformed to absorbance. However, this method selected wavelengths specific to the species studied [5,29,48,49,50], whereas the present study used a faster and simpler method that simultaneously collected both reflectance and transmittance data using contiguous hyperspectral bands in the visible spectrum (400–700 nm). Other studies [6] resulted in the collection of 300 hyperspectral coefficients with a resolution of 1 nm and a measurement time of less than 5 s [6]. These coefficients obtained through multivariate modelling provide a more accurate analysis, as they consider small contributions from each wavelength rather than selecting a specific band or predetermined spectral range for a specific species [23,28,30,31,51].

The use of integrating spheres to simultaneously collect T and R data may result in significant error if the sample positioning is not perfectly aligned, causing a fraction of the transmitted light to not reach the integration surface [25]. However, coupling two sensors can effectively solve this issue, as demonstrated in previous studies [6,8,19]. Our data suggest that the best results for pigment estimation can be obtained using absorbance data (Table 1 and Figure 12). By observing the scatterplots for each calibration, cross-validation, and prediction phase between chlorophylls (Figure 9A–F and Figure 12A–F) and carotenoids (Figure 9G,H and Figure 12G,H) using reflectance (Figure 10) and absorbance (Figure 12), it is demonstrated that the absorbance data consistently yield more robust parameters (Table 1).

Absorbance spectra are more informative for PLSR analysis than reflectance spectra because absorbance is influenced by the molecular composition of the samples [2,52]. In particular, the absorbance signal is affected by various molecular interactions, such as those involving hydrogen bonding, van der Waals forces, and dipole-dipole interactions, as well as the presence and concentration of chlorophylls and carotenoids [53,54]. Therefore, changes in the molecular composition, including the levels of specific pigments or other functional groups, can produce significant variations in the absorbance spectra.

The reflectance of light is mostly influenced by surface reflectivity and is not as affected by molecular interactions [6,14,24,55]. In addition, the use of PLSR analysis on absorbance data has shown comparable or even higher correlations compared to other multivariate methods, such as principal component analysis or linear discriminant analysis, as used in plant studies [8,22,30,35].

The findings of this study indicate that using absorbance data is more effective in estimating pigments than reflectance data. This is supported by the higher values for the Offset, RMSE_CV_, RMSE_P_, and RPD metrics, as shown in Table 1. The evidence for this is seen in the low RMSE_C_ values, high correlation coefficients (R^2^ values of 0.88 and 0.91 for absorbance and 0.80 and 0.78 for reflectance), and low Bias values of the absorbance data. Additionally, the RPD values for absorbance data were found to be 1.6 to 2.5 times higher than those for reflectance data, demonstrating the superior measurement potential of absorbance data. These results align with previous studies, such as those by D’Acqui et al. (2010) [56] and Oliveira-Júnior et al. (2020) [57]. While the reflectance data also showed good parameter estimation capabilities, the RPD values for absorbance were higher (10.3 to 21.7) than those for reflectance (6.1 to 9.6) (Table 1). Accordingly, our results support the use of absorbance data for estimating pigments in plants.

#### 3.2.2. Cross-Validation to Chloroplastidic Pigments

The results of the cross-validation (RMSE_CV_) tests indicated that the absorbance data were more effective in modelling chloroplast pigments than the reflectance data, similar to the calibration (RMSE_C_) phase. The correlation between the predictor variables (absorbance or reflectance) and the predicted variables (photosynthetic pigments) was stronger for absorbance data than for reflectance data (Figure 9, Figure 10, and Figure 12). The red dots in the figures represent the cross-validation results of the multivariate statistics. The coefficients of determination for Chl *a*, Chl *b*, Chl *a*+*b*, and Car were similar in the cross-validation step (Table 1), but lower values were recorded for absorbance data compared to reflectance data (Figure 10, blue dots) and even compared to the calibration step (Figure 12, blue dots). However, the results for carotenoids were exceptional, with “r” values of 0.86 and 0.92 for reflectance and absorbance, respectively (Table 1 and Figure 12G-H) and compared to these studies [6,8]. In this sense, hyperspectral sensors and PLSR analysis of absorbance data have shown a high potential for model generation, particularly in the visible (VIS) bands, as exemplified by their application in maize plants [23,30,50,58].

### 3.3. Relationship between Pigment Concentration and Reflectance and Absorbance for Leaves

The scatterplot analysis (Figure 10, Figure 11 and Figure 12) demonstrates that the Chl *a*, Chl *b*, and Chl *a*+*b* reflectance data have values that are further from the 1:1 line compared to absorbance data (Figure 12). This difference can be attributed to variations in plant development, specifically SLA and LAI, which impact pigment distribution in leaves, as well as variations in leaf thickness that were not removed during model development and reflect the true pigment composition and content in thylakoid membranes [10]. The range of Chl *a* (0.13 to 0.46 g m^−2^) and Chl *a*+*b* (0.19 to 0.65 g m^−2^) concentrations in tobacco plants and maize [30,59] highlights the significance of the variation. Despite the small variations in Chl *b* (0.058 to 0.19 g m^−2^) and Car (0.033 to 0.16 g m^−2^) concentrations, carotenoids showed better relationships due to their low data dispersion around the average (Figure 8, Figure 10, and Figure 12), as estimated by PLSR [36,56]. Although other models may exhibit a similar or better correlation with reflectance data [8,60], the combination of absorbance data and PLSR demonstrated superior correspondence and linearity from hyperspectral data for chlorophyll estimation.

The initial hypothesis is supported by the fact that absorbance (A) is not influenced by transmittance (T) and reflectance (R), but the other way around; T and R are impacted by the material’s intrinsic absorption. The presence of chloroplast/photosynthetic pigments [10] or extrachloroplastidic pigments [6] confirms that A is responsible for absorbing light and determining the absorption-interaction-pigment spectra and hyperspectral signatures, resulting in a more accurate prediction of pigments that closely resembles their natural characteristics. The Lambert—Beer law assumes a homogeneous, clear, and low optical density solution but does not accurately apply to nonhomogeneous materials, such as biological materials including leaves [61]. Therefore, reflectance and transmittance do not have the ability to absorb light and may either overestimate or underestimate the presence of pigments in plant samples. For example, if reflectance data were perfectly complementary to absorbance, the ratio would be 1:1, but different pigments and structural changes in leaves result in this relationship being more strongly correlated with peaks at 435 and 674 nm (Figure 11A,C) and weaker at 550 nm (Figure 11B) [8,10], with a statistically significant relationship (*p* < 0.001) (Figure 11). As a result, green bands may not be significant in some analyzes, but they still correspond to changes resulting from leaf pigments.

### 3.4. Optical Characteristics for Predicting Carotenoids

The results showed that estimating carotenoid concentration using PLSR and hyperspectral response is difficult due to the overlapping of various pigments and components. However, using contiguous hyperspectral bands and the PLSR technique resulted in more accurate, robust, and dependable models (r = 0.922; *p* < 0.001). The absorption-based approach, proposed by Gitelson and Solovchenko (2018) [25], can improve carotenoid estimation, particularly in the blue—green region. This interaction of light with leaves improves our understanding of light absorption properties and creates new opportunities for plant physiology and photobiology.

Our recent research and new method enable the evaluation of reflectance and absorbance curves in complex in vivo systems [5,6,27]. Analysis with multivariate techniques provides a better understanding of the plant’s state, such as whether it is suffering from a nutritional deficiency, oxidative damage, photoinhibition of photosynthesis, damage from cold or heat, or injury from pests and insects [47,62,63]. In terms of plant productivity, absorbance curves allow us to estimate the amount of light energy plants can absorb without hindering the photochemical step of photosynthesis, depending on the light intensity, quality, or duration of each measure.

Accurately estimating the levels of chlorophyll and carotenoids is crucial for understanding plant health and productivity, as these pigments play essential roles in photosynthesis and other physiological processes [28,30]. Combining hyperspectral measurements and predictions from two sensors has shown promise in improving the detection of yellow rust on winter wheat, with a high accuracy of up to 94–95% for the fluorescence method [59]. However, this approach can provide a more comprehensive and accurate assessment of plant health by capturing both structural and physiological information as well as in other agronomic plants [29,48,64]. Moreover, detecting plant diseases at an early stage can help reduce crop losses and increase yields. Therefore, further research is needed to optimize the use of hyperspectral sensors for disease detection and assess their potential for wider applications in precision agriculture [24,65,66].

The distribution of carotenoids that closely resembles the in vivo system allows for the prediction of other photochemical parameters, such as the xanthophyll cycle and electron transport rate, efficient light utilization by plants, nitrogen incorporation, energy dissipation efficiency, and photoprotection rates through mainly green and yellow bands (525–580 nm) [62,67], which are among the most significant differences in spectral signatures (Figure 7, Figure 8, Figure 9, Figure 10, Figure 11 and Figure 12) [10].

### 3.5. Prediction Based on an Independent Data Set

The models were evaluated using a different spectral data set, which was not used for calibration or validation. The results, displayed as green dots in Figure 10 and Figure 12, aimed to enhance our understanding of the interaction between light, the calibrated models, for chlorophylls and carotenoids.

Figure 12 shows that the prediction of Chl *a*, Chl *a*+*b*, and Car was more accurate when using absorbance data, than when using reflectance data, as demonstrated in Figure 10. The pigments estimated using the PLSR technique in conjunction with hyperspectroradiometric curves were found to be in agreement with those determined in the laboratory using traditional techniques such as spectrophotometry, spectroscopy techniques, and ^1^H-NMR or UHPLC [8]. The coefficients of determination for Chl *a*+*b* and Car were similar to those reported by Saad et al. (2017) [68], who used PLSR to estimate high correlations (R^2^ ≥ 0.82) for tomatoes using bands in the VIS/NIR region. Although the coefficients of determination and correlation for Chl *a* and Chl *a*+*b* were moderate (with R^2^ values of 0.041 and 0.057), they were still lower than those reported in other studies [22,68,69]. These authors reported that components of the epidermis contributed to the poor quality of the generated models, including the effects of moisture, surface modifications, waxes, epicuticular layers, as well as the presence of trichomes and stomata [22,68,69,70,71]. The distribution and organization of concentration pigments in leaves may also influence the extinction or attenuation coefficients of light, which could help explain the observed correlation [72,73].

The study found a strong correlation between the absorbance and reflectance data and the distribution of pigments in leaves, as indicated by RPD values greater than 4.7 for absorbance data and 4.5 for reflectance data (Table 1, Figure 10 and Figure 12). The models for predicting Chl *a*+*b* and Car concentrations were accurate and had excellent correlations in the spectral range (0.2 to 0.6 g m^−2^ for Chl *a*+*b* and 0.04 to 0.16 g m^−2^ for Car, Figure 7, Figure 9, Figure 10, and Figure 12). The absorbance data based on two sensors were considered to better reflect the relationship between light and pigments in the thylakoid membranes, being closer to in vivo events [10,42,74]. The cross-validation estimate for the reflectance data was also found to be better than the estimate using an independent data set. Therefore, high values for Chl *a* and Chl *a*+*b* were also observed in previous steps.

The prediction of leaf pigment concentration in the validation set (as indicated by the green dots) suggests a lower accuracy compared to the prediction in the calibration set, as indicated by the SEP values [75]. Despite this, the results still exhibit a higher level of accuracy for Chl *a*+*b*, compared to data obtained through PLSR. These findings demonstrate the potential of combining hyperspectral data with PLSR for the study of leaf pigments [10,42,74]. Most previous studies have only focused on reflectance curves, limiting their ability to fully comprehend the spectral interactions that lead to accurate pigment estimation. In this way, the method provides a more efficient and cost-effective alternative to laboratory methods, which can be both time-consuming and potentially harmful to the environment [22,68,69,70,71]. The use of two sensors and PLSR analysis represents a significant advancement in the field, enabling a quick and accurate estimation of carotenoids, anthocyanins, flavonoids, and phenolic compounds in tobacco and lettuce, plants with varying pigment contents, and leaf structures [75].

### 3.6. Hyperspectral Two-Sensor and PLSR Analysis Are Good Tools to Predict Pigments and Understand Profile Optical Properties

The integration of high-resolution sensors for spectral acquisition and analysis using curve deconvolution and PLSR [76] along with other multivariate techniques [6,24] has resulted in the development of more accurate and robust models for predicting pigment concentrations. The use of contiguous spectral bands instead of infrared spectra significantly improved the models, as demonstrated by the lower RMSE values. The ability to choose between different spectral bands through discriminant analysis also contributes to the precision and accuracy of high-resolution spectral data (Figure 10 and Figure 12).

While reflectance spectra remain a popular method for agricultural monitoring [36,77] and environmental data collection due to their quick and remote nature, the true understanding of leaf optical profile absorption properties can only be obtained through absorbance spectra deconvolution [6].

The use of leaf optical models, such as the PROSPECT model [78], has been crucial for understanding the optical properties of leaves and estimating photosynthetic pigment concentrations [46,79]. However, previous models have not incorporated photoprotective pigments, for example, carotenoids based on an absorbance [A = 1 − (R + T)] [6], in these models, which are important indicators of plant physiological and ecological functions. The development of PROSPECT-MP^+^ [46] has addressed this limitation by including the contributions of both photosynthetic and photoprotective pigments in the leaf spectrum [46,59,79].

Furthermore, the distribution of carotenoids in the spectral signatures closely resembles the in vivo system [24], allowing for the prediction of other photochemical parameters, such as the xanthophyll cycle and electron transport rate [80,81]. This information is critical for understanding plant efficiency in utilizing light and incorporating nitrogen, as well as for assessing energy dissipation efficiency and photoprotection rates.

Accordingly, the simultaneous measurement of both the adaxial and abaxial faces of leaves using spectroradiometers [8,10] has further reinforced the importance of using robust data to better understand the optical leaf profile and estimate major chloroplastidic pigments in leaves [8]. Therefore, the results of this study suggest that absorbance spectral curves with higher reliability can be obtained through two sensors and PLSR statistical routines [36,82].

## 4. Conclusions

Our study demonstrates the potential of using two hyperspectral sensors to collect both reflectance and absorbance data, combined with PLSR, as a powerful tool for predicting in vivo photosynthetic pigments. The results showed strong correlation coefficients between the predicted parameters and hyperspectral absorbance data, particularly for carotenoids. Specifically, carotenoids exhibited high and significant correlation coefficients using the PLSR method, with R^2^_C_ = 0.91, R^2^_CV_ = 0.85, and R^2^_P_ = 0.90. These findings present promising opportunities for future research in plant development and pigment prediction. However, future studies should consider the variability of plant samples from different botanical families to establish a wider range of results. Additionally, this approach highlights the need for further research that takes into account not only the variation in chlorophylls and carotenoids, but also the biochemical composition of leaves.

## Figures and Tables

**Table 1:** Statistical metrics from the PLSR model in the calibration, cross-validation, and predicted phases. Pearson correlation (r), model goodness-of-fit (R^2^), slope, offset, root mean squared error (RMSE), ratio of performance to deviation (RPD) and ad Bias to base models (a prediction using an independent sample coupled to calibrated models), parameters of chlorophylls and carotenoids, parameters from reflectance (single sensor), and absorbance (two sensors) hyperspectral data of tobacco leaves. The bold numbers represent statistically significant PLSR parameters.

Sensors	PLSR Models	Parameters	PLSR Parameters						
			r	R^2^	Slope	Offset	RMSE	RPD	Bias
**Reflectance** – **Single sensor**	**Calibration**	Chl *a* (g m^2^)	0.88	0.77	0.78	0.06	0.03	2.09	-
		Chl *b* (g m^2^)	0.84	0.70	0.70	0.03	0.01	1.82	-
		Chl *a*+*b* (g m^2^)	0.88	0.78	0.80	0.07	0.03	2.13	-
		Car (g m^2^)	0.90	0.80	0.79	0.02	0.01	2.14	-
	**Cross-Validation**	Chl *a* (g m^2^)	0.86	0.75	0.76	0.06	0.03	1.98	-
		Chl *b* (g m^2^)	0.81	0.65	0.67	0.03	0.01	1.70	-
		Chl *a*+*b* (g m^2^)	0.85	0.72	0.78	0.08	0.04	1.89	-
		Car (g m^2^)	0.87	0.76	0.76	0.02	0.01	2.03	-
	**Prediction**	Chl *a* (g m^2^)	0.80	0.65	0.64	0.09	0.04	1.68	0.002
		Chl *b* (g m^2^)	0.76	0.58	0.74	0.02	0.02	1.54	0.002
		Chl *a*+*b* (g m^2^)	0.78	0.61	0.67	0.12	0.05	1.60	0.001
		Car (g m^2^)	0.85	0.73	0.78	0.02	0.01	1.93	0.000
**Absorbance** – **Two sensors**	**Calibration**	Chl *a* (g m^2^)	**0.94**	**0.88**	0.88	**0.03**	**0.02**	**2.89**	-
		Chl *b* (g m^2^)	**0.90**	**0.82**	0.82	**0.02**	**0.01**	**2.33**	-
		Chl *a*+*b* (g m^2^)	**0.93**	**0.87**	0.87	**0.04**	**0.03**	**2.77**	-
		Car (g m^2^)	**0.95**	**0.91**	0.91	**0.01**	**0.01**	**3.31**	-
	**Cross-Validation**	Chl *a* (g m^2^)	**0.89**	**0.79**	0.88	**0.05**	**0.02**	**2.20**	-
		Chl *b* (g m^2^)	**0.87**	**0.75**	0.77	**0.02**	**0.01**	**2.00**	-
		Chl *a*+*b* (g m^2^)	**0.89**	**0.79**	0.82	**0.06**	**0.04**	**2.18**	-
		Car (g m^2^)	**0.93**	**0.85**	0.87	**0.01**	**0.01**	**2.67**	-
	**Prediction**	Chl *a* (g m^2^)	**0.83**	**0.69**	0.76	**0.06**	**0.04**	**1.80**	0.000
		Chl *b* (g m^2^)	**0.80**	**0.64**	0.80	**0.01**	**0.02**	**1.67**	0.000
		Chl *a*+*b* (g m^2^)	**0.79**	**0.62**	0.77	**0.08**	**0.06**	**1.63**	0.000
		Car (g m^2^)	**0.95**	**0.90**	0.86	**0.01**	**0.01**	**3.16**	0.000

**Footnote:** Chlorophyll *a* (Chl *a*), Chlorophyll *b* (Chl *b*), Chlorophyll total (Chl *a*+*b*), Carotenoids (Car) expressed by area units. RMSE_C_ (calibration phase), RMSE_CV_ (cross-validation phase), RMSE_P_ (prediction phase).

## Data Availability

Not applicable.
